# “When you leave your country, this is what you’re in for”: experiences of structural, legal, and gender-based violence among asylum-seeking women at the Mexico-U.S. border

**DOI:** 10.1186/s12889-023-16538-2

**Published:** 2023-09-02

**Authors:** Kaylee Ramage, Emma Stirling-Cameron, Nicole Elizabeth Ramos, Isela Martinez SanRoman, Ietza Bojorquez, Arianna Spata, Brigitte Baltazar Lujano, Shira M. Goldenberg

**Affiliations:** 1 Division of Epidemiology and Biostatistics, School of Public Health, San Diego State University, San Diego, CA USA; 2 Centre for Gender and Sexual Health Equity, Faculty of Medicine, University of British Columbia, Vancouver, Canada; 3 Al Otro Lado, Tijuana, Baja California Mexico; 4 Center for Latin American Studies, San Diego State University, San Diego, CA USA; 5 Department of Population Studies, El Colegio de la Frontera Norte, Tijuana, Mexico; 6 Division of Infectious Diseases and Global Public Health, School of Medicine, University of California, San Diego, USA

**Keywords:** Gender-based violence, Migration, COVID-19, Structural violence, Immigration policy, Asylum deterrence

## Abstract

**Background:**

Recent U.S. immigration policy has increasingly focused on asylum deterrence and has been used extensively to rapidly deport and deter asylum-seekers, leaving thousands of would-be asylum-seekers waiting indefinitely in Mexican border cities, a large and growing proportion of whom are pregnant and parenting women. In the border city of Tijuana, Mexico, these women are spending unprecedented durations waiting under unsafe humanitarian conditions to seek safety in the U.S, with rising concerns regarding increases in gender-based violence (GBV) among this population during the COVID-19 pandemic. Given existing gaps in evidence, we aimed to describe the lived experiences of GBV in the context of asylum deterrence policies among pregnant and parenting asylum-seeking women at the Mexico-U.S. border.

**Methods:**

Within the community-based *Maternal and Infant Health for Refugee & Asylum-Seeking Women (MIHRA)* study, we conducted semi-structured qualitative interviews with 30 asylum-seeking women in Tijuana, Mexico between June and December 2022. Eligible women had been pregnant or postpartum since March 2020, were 18–49 years old, and migrated for the purposes of seeking asylum in the U.S. Drawing on conceptualizations of structural and legal violence, we conducted a thematic analysis of participants’ experiences of GBV in the context of asylum deterrence policies and COVID-19.

**Results:**

Pregnant and parenting asylum-seeking women routinely faced multiple forms of GBV perpetuated by asylum deterrence policies at all stages of migration (pre-migration, in transit, and in Tijuana). Indefinite wait times to cross the border and inadequate/unsafe shelter exacerbated further vulnerability to GBV. Repeated exposure to GBV contributed to poor mental health among women who reported feelings of fear, isolation, despair, shame, and anxiety. The lack of supports and legal recourse related to GBV in Tijuana highlighted the impact of asylum deterrence policies on this ongoing humanitarian crisis.

**Conclusion:**

Asylum deterrence policies undermine the health and safety of pregnant and parenting asylum-seeking women at the Mexico-U.S. border. There is an urgent need to end U.S. asylum deterrence policies and to provide respectful, appropriate, and adequately resourced humanitarian supports to pregnant and parenting asylum-seeking women in border cities, to reduce women’s risk of GBV and trauma.

## Introduction

Gender-based violence (GBV) refers to harm perpetrated against individuals or groups based on their gender, and includes physical, sexual, or mental harms, threats, coercion, or other impacts on freedom and autonomy [1, 2]. GBV is rooted in the power structures and sociocultural ideologies of society and is perpetuated by gender inequalities, gendered norms, and abuses of power [1, 2]. Survivors of GBV face both short- and long-term consequences, including adverse physical health outcomes (e.g., sexually transmitted infections, unintended pregnancy, death) and mental health outcomes (e.g., depression, post-traumatic stress disorder, anxiety, suicidal ideation and substance use disorders) [3–7].

GBV is a significant threat to women throughout their migration journeys, particularly those who have been forcibly displaced, such as asylum-seekers [5]. GBV represents an additional layer of vulnerability which intersects with other forms of marginalization for migrant women including racism, legal status, language, and pregnancy or parenting status [8]. For women from countries with high levels of gender inequality, such as Honduras, Guatemala, and El Salvador (i.e., the Northern Triangle), GBV represents a key driver of migration to the U.S. to seek asylum [9]. Women transiting through Mexico face high rates of exposure to GBV, with a 2019 study finding that almost one quarter of migrating women experiencing some form of violence and of those experiencing violence, 52.8% experienced psychological violence, 10.8% experienced kidnapping, 53.8% experienced theft, 22.6% experienced physical assault, and 14.2% experienced rape [10].

Previous reports among migrant women have documented experiences of GBV throughout the migration pathway (i.e., pre-migration, in transit, at the border, and in receiving countries) [2, 11] and among women waiting at the Mexico-U.S. border [12]. However, despite the recognition that asylum-seeking women are, as a whole, highly vulnerable to GBV and the knowledge that structural factors can contribute to this vulnerability [13], few studies have examined GBV among pregnant and parenting asylum-seekers affected by the structural and legal violence of asylum deterrence policies at the Mexico-U.S. border [14, 15], especially since the 2020 implementation of Title 42 U.S.C. Section 265 of the 1944 Public Health and Service Act (‘Title 42’).

In the last several years, the focus of the U.S. asylum system has shifted from detention and deportation to expulsion and exclusion [16], with an unprecedented rise in asylum deterrence policies, including the Migrant Protection Protocol (MPP) (i.e., where those seeking asylum at the Mexico-U.S. border are returned to Mexico to await U.S. immigration court hearings, rather than being allowed entry to the U.S. during this period), Metering (i.e., where an artificial limit has been placed on the number of people seeking asylum who are processed at ports of entry) [17], Zero Tolerance (i.e., where asylum-seeking parents were separated from their children, with limited information about each other’s whereabouts and safety, and often deportation of the parents to their countries of origin without their children) [18], and safe third country agreements such as Asylum Cooperative Agreements (i.e., where people seeking asylum are arbitrarily blocked from requesting humanitarian protection in the U.S. and are sent to other potentially unsafe third countries instead) [19, 20]. Humanitarian parole has also been inconsistently applied for asylum-seekers to the U.S., limiting its usefulness for pregnant and postpartum asylum-seekers. More recently, under the guise of COVID-19 mitigation, Title 42 has been used to restrict entry to over one million asylum-seekers and refugees at the U.S. border between March 2020 [16] and May 2023, and has been replaced by a resurrected Trump-era Transit Rule, where migrants who fail to obtain an appointment to be processed through the smartphone application CBPOne, and who have failed to seek asylum in a country through which they transited, are later deemed ineligible for asylum in the U.S. [21, 22]. This new policy is also complicated by the forced use of the CBP One smartphone application for processing appointments, an app which is limited in its accessibility, functionality, and appointment availability [23, 24]. These policies have built on the U.S’s exclusionary and xenophobic history towards migrants and stand in sharp contrast to the U.S.’s supposed commitment to upholding the right to asylum under the 1980 Refugee Act, the 1951 Refugee Convention, and the 1967 Protocol relating to the Status of Refugees, the Convention Against Torture [25–27].

The focus on asylum deterrence has allowed U.S. Customs and Border Protection (CBP), the border enforcement arm of the Department of Homeland Security, to limit asylum-seeking, forcing would-be asylum-seekers to wait along the border for designated “legal” opportunities to cross the border and denying them access to humanitarian protections and supports [28], leading to unprecedented backlogs (e.g., over two million pending cases as January 2023) [29] and an average stay of over 12 months for asylum seekers in Tijuana [30].

Current U.S. migration policies restricting access to the asylum process for migrants at the Mexico-U.S. border have contributed to the humanitarian crisis for those waiting in border cities, such as Tijuana, including egregious gaps in basic services and supports and to health inequities for migrant women and children [21]. These restrictive immigration policies interact with other socio-structural processes including racism, gender inequity, and xenophobia to increase asylum-seeking women’s risk of GBV during their immobility at the Mexico-U.S. border, and ultimately act as a form of both structural and legal violence. Structural violence refers to violence perpetuated through the social relations and power dynamics that shape how individuals and groups interact within a social system (e.g., economic, political, legal, religious, and/or cultural systems) and results in sustained inequities by marginalizing people and communities [31]. Legal violence, in turn, focuses on the “various, mutually reinforcing forms of violence that the law makes possible and amplifies” [32] (p. 1384). Analysis of legal violence allows for investigation of contexts created by contradictory policies and laws such as Title 42 which, in name, purports to increase public health and public safety but, in fact, results in harm.

Although prior evidence demonstrates that migrant women face increased vulnerability to GBV, there remains a substantial gap in empirical evidence regarding the impact of asylum deterrence policies on migrant women’s experiences of GBV. Given existing gaps in evidence, we aim to describe the lived experiences and impacts of GBV in the context of structural and legal violence enacted through asylum deterrence policies on pregnant and parenting asylum-seeking women at the Mexico-U.S. border during the COVID-19 pandemic.

## Methods

In this qualitative inquiry, we explore the far-reaching impact of structural violence through asylum deterrence policies on the experiences of GBV for pregnant and postpartum asylum-seeking women at the Mexico-U.S. border since the beginning of the COVID-19 pandemic (March 2020). This research was conducted as part of the *Maternal and Infant Health of Refugee and Asylum-Seeking Women* (MIHRA) Study, a mixed-methods, community-based research study aimed at documenting the socio-structural determinants of maternal and infant health among pregnant and parenting asylum-seeking women at the U.S.-Mexico border. All aspects of the study were conducted in close collaboration with Al Otro Lado (AOL), a non-profit, community-based organization that provides holistic legal and humanitarian support to refugees, deportees, asylum-seekers, and other migrants in the U.S. and Tijuana, Mexico through a multi-disciplinary, client-centered, harm-reduction practice.

### Study setting

Tijuana is a city of approximately two million people in northwestern Mexico. Given its proximity to San Diego, California and its location along the Mexico-U.S. border, Tijuana is an international migration hub, with the San Ysidro Port of Entry (between San Diego and Tijuana) being the world’s busiest land border crossing. The exact number of asylum-seekers in Tijuana is unknown and ever-changing; however, it is estimated that, as of May 2023, approximately 14,000 to 16,000 migrants are staying in Tijuana until they have the chance to cross into the U.S. [33]. Since the start of the COVID-19 pandemic and the implementation of Title 42, the number of migrants waiting in border cities, including Tijuana, has been increasing, many of whom are forced to wait for months or years in deplorable humanitarian conditions, such as overcrowded and unsanitary shelters with limited access to safe and sufficient food and water. This influx of migrants amidst Title 42 has caused further strain to health and social systems which were already overwhelmed due to the COVID-19 pandemic [34, 35].

Furthermore, pregnant and parenting asylum-seekers in Mexico face other barriers to their health and well-being. While Mexico has extended free access to public health services to all persons in the country, including migrants, gaps and barriers to services still exist [36, 37] and many migrants still have to pay out-of-pocket for private care or for medications, laboratory services, or other services within the public healthcare system [37]. Many migrants have reported that affordability and the (lack of) ability to pay act as barriers to accessing care, including sexual, reproductive, and pregnancy-related care. These barriers were exacerbated during the COVID-19 pandemic due to strained health and social systems and pandemic-related closures [36–38].

### Data collection

Eligible participants were: self-identified women; ages 18–49 years; experienced one or more pregnancies or been postpartum^1^ and had migrated to Tijuana, Mexico to seek asylum in the U.S since the beginning of the COVID-19 pandemic (March 2020); and were able to provide informed consent. Participants were recruited by a team of trained, multilingual, community-based staff at AOL and SDSU led community-based recruitment via study posters, word-of-mouth, direct referral, and outreach. Women were purposively sampled to reflect diverse experiences, including migration history (e.g., place of origin, transit history, length of time in Tijuana), healthcare experiences (e.g., prenatal care, labor and delivery, postpartum care), and age (e.g., younger vs. older women).

Following informed consent, in-depth, semi-structured interviews were conducted with women in their primary language (Spanish or Haitian Creole) in private offices at the AOL Tijuana office from June-December 2022. Data collection was conducted by a team of trauma-informed, multilingual, and multidisciplinary staff at AOL and SDSU, most of whom had lived migration and/or extensive community experience. Interviews in Haitian Creole were facilitated with the support of a trained, multilingual interpreter. A brief demographic survey was administered before the interview to collect relevant sociodemographic information. Interviews were audio-recorded and took approximately 60 to 90 min. Interviews followed an open-ended interview guide which explored migration experiences (e.g., reason for migration, migration history, issues with documentation, immigration enforcement), experiences with violence (e.g., GBV, kidnapping, threats, extortion), and pregnancy experiences (e.g., healthcare access and navigation, labor and delivery experiences, discrimination, contraception, perinatal health). Participants received $30 USD for their participation, time and travel.

### Data analysis

Interviews were transcribed verbatim, translated, and accuracy checked and de-identified. Thematic data analysis involved an iterative process of reading, discussing, and coding amongst members of the research team in close collaboration with our community partners. This team-based, collaborative approach supported rigor by ensuring that team member’s interpretations of the data were “checked” by other members of the team, and that analyses incorporated the deeply nuanced insights of our community partners working at the frontline of humanitarian aid for asylum-seeking women and their families. Analyses involved both inductive and deductive approaches. First, an initial coding framework was developed based on our interview questions, and the key themes that emerged through reading and re-reading of transcripts. Codes were iteratively revised as data collection and analyses progressed. In the final stage of analysis, we drew on a conceptual structural violence framework [31, 39] and the available literature related to asylum-seeking, asylum deterrence policies, and GBV to describe the lived experiences of GBV in the context of asylum deterrence policies among pregnant and parenting asylum-seeking women at the Mexico-U.S. border.

## Results

Of the 30 pregnant and parenting asylum-seeking women who participated, on average, women were 29 years of age and had 2.5 children (Table 1). Twelve (40%) were internal migrants from Mexico, 13 (43%) were from Northern Triangle countries of Central America (i.e., El Salvador, Honduras, and Guatemala), and 5 (16.7%) were from Haiti. Most identified as being of Hispanic, Latino, or Spanish origin, 23.3% as Black, and 27.6% as Indigenous. On average, the participants had been in Tijuana for an average of 10.7 months, ranging from 1 month to 6 years.

**Table 1:** Demographic Characteristics of Pregnant and Parenting Asylum-Seeking Women in Tijuana, MIHRA Study, 2022 (*N* = 30)

**Characteristic**		**Total/Mean (% or Range)** ***N* = 30**
**Age**	*Mean (range)*	29.1 years (18 years – 41 years)
**Number of Children**	*Mean (range)*	2.5 children (1 child – 5 children)
**Country of Origin**	*Mexico*	12 (40.0%)
	*El Salvador*	3 (10.0%)
	*Honduras*	7 (23.3%)
	*Guatemala*	3 (10.0%)
	*Haiti*	5 (16.7%)
**Of Hispanic, Latino, or Spanish Origin**		26 (86.7%)
**Identify as Black**		7 (23.3%)
**Identify as Indigenous**		8 (27.6%)
**Duration of Time in Tijuana**	*Mean (range)*	10.7 months (1 month—6 years)

Participants’ narratives emphasized four inter-related themes related to the GBV that they had experienced along their migration journey that were compounded by the impacts of asylum deterrence policies: 1) perpetuation of GBV across the migration journey; 2) asylum-seeking women’s vulnerability to GBV as exacerbated by asylum deterrence policies during COVID-19; 3) negative physical and mental impacts related to GBV; and 4) lack of access to responsive justice and violence-related supports.

### Perpetuation of gender-based violence across the migration journey

In our study, women experienced GBV in their country of origin, in-transit to Tijuana, and in Tijuana while waiting to seek asylum. This continuum of GBV between countries of origin and Tijuana included physical violence, sexual violence, kidnapping, and threats of violence and reflects the myriad of gendered dangers, whether real or perceived, that women experienced before and throughout their migration. Asylum deterrence policies forced women to wait in these unsafe conditions, perpetuating their experiences of GBV and creating conditions that increased women’s vulnerability to GBV, ultimately allowing its existence in their lives. None of the women in our study described having had access to the humanitarian parole process, even if they would have been eligible, which also forced them to give birth in sometimes unsafe and discriminatory settings, putting them at further risk of violence, harm, and adverse perinatal outcomes.

These conditions left women vulnerable to the inevitability of GBV along their migration journey, with others recognizing the lack of impunity for perpetrators of GBV. One woman described an experience with a truck driver who had given her and a friend a ride and who had tried to extort her sexually as payment. He told them, “*when you leave your country, this is what you’re in for*” (Asylum-seeking woman from El Salvador, Age 21 years), speaking to the pervasive societal view that GBV was normal and was something that women were meant to endure. Another woman mentioned that experiences of abuse are “*things that happen and that one has to overcome as well”* (Asylum-seeking woman from Honduras, Age 26 years*).*

#### Pre-migration

All women in our study described fleeing their home country due to the threat of violence for themselves and/or their families, including extortion, physical violence, sexual violence, or (attempted) kidnapping. Several women described the constant threat of violence and attempts at extortion from cartels or gangs in their neighbourhoods

*“I left my place of origin because of violence, because of threats, because I was kicked out of the place where I lived. Armed people came to my house at night and threatened to kill me and my children. I am a single mother, I have three children and at that moment, as a mother, all I was thinking about was protecting my children. So I grabbed my children, as much as I could and ran out of there, running away, and made it all the way here to Tijuana*.” (Asylum-seeking woman from Mexico, 26 years old)

Another woman described the attempted kidnapping of her daughter, alongside continued threats and extortion. This participant described grappling with the decision to flee her home country and leave her extended family behind: “*Coming to a place, not because you want to leave, but because you are forced to leave*” (Asylum-seeking woman from Guatemala, 23 years old).

#### In transit from their home country to Tijuana

During their migration from their home country to the Mexico-U.S. border, women were highly vulnerable to GBV and many experienced sexual and physical violence. This vulnerability was exacerbated by the lack of safe spaces for migrants including unsafe living conditions, exploitative working conditions, and financial precarity, where they are constantly are at real or perceived risk of harm. For women who did not have the financial means for a flight to Tijuana, the long journey from their home country often necessitated paying others for transport, stopping for short-term work in new places, and then moving on after weeks or months. Many of the women were desperate to find resources to meet their basic needs and, due to their situation, were vulnerable to financial or sexual extortion. One woman was robbed during her migration journey and was forced to ask for help from others to get the resources she needed to continue her journey.

*“[They] attacked us and they stole the money I was carrying… I was robbed and here he holds up the gun to my head, he takes off all my clothes to see if I have more money. He left me without money. I was on the street asking for help to continue the journey.”* (Asylum-seeking woman from Haiti, 32 years old)

#### In Tijuana

With the addition of Title 42 to existing asylum deterrence policies and the closure of the Mexico-U.S. border to most asylum-seekers in March 2020, many women found themselves living in Tijuana for months as they waited for a legal opportunity to cross. As there was minimal movement across the border, inordinate pressures were placed on existing resources (including housing, food, employment, and humanitarian aid), creating inhospitable conditions in Tijuana and other border cities and essentially trapping women in a system which perpetuated the violence and harms against them rather than providing them support or refuge and thus perpetuating legal violence.

Although they had expected to reach safety, with one woman sharing that she decided to migrate through Tijuana because it was the “*farthest*” from the violence in her city (Asylum-seeking woman from Mexico, Age 26 years), women continued to experience GBV in Tijuana, including pregnancy-related discrimination, physical violence, and sexual violence. One woman described her experience with an abusive partner: “*And then he kicked me out, he hit me, he did whatever he wanted. When I arrived [to the shelter for pregnant women], I was all beaten up and that’s where they took care of me and my baby*” (Asylum-seeking woman from Honduras, 26 years old). This was perpetuated by the humanitarian crisis at the Mexico-U.S. border caused in part by the implementation of asylum-deterrence policies.

### Asylum deterrence policies: exacerbating vulnerability to gender-based violence

Asylum-seekers, by definition, are seeking safety. Women’s experiences clearly reflected how structural and legal violence were perpetuated by the impact of U.S.-based asylum deterrence policies on the (un)availability of support, resources, and basic needs in Tijuana, where existing humanitarian aid was inadequate to meet the needs of the influx of migrants. For women in our study, the humanitarian crisis in Tijuana engendered by the addition of Title 42 to existing asylum deterrence policies severely elevated risk of harm due to the lack of safe spaces, with few available supports, ultimately increasing their risk and perpetuating their experiences of GBV.

Asylum-seeking women routinely reported trying to find safe space for themselves and their young children in overcrowded shelters, many spending time sleeping outside, under bridges, or waiting in line for days. Surprisingly, while pregnant women or women with children in humanitarian emergencies are often prioritized for housing and other resources, in this study, several women described facing discriminatory treatment and being refused services due to being pregnant, having children, and/or on the basis of their migration status or race.

“*Nobody wanted to open their door for us. We walked all over the streets of Tijuana with our children and our backpacks, and they said it was closed, that they didn’t want families. And we searched and searched and until now, we are in [name of shelter]. And they haven’t taken us in, they have us outside. We are waiting… being cold and under the rain. I’ve gone out to look for a shelter and no one wants to receive us. And we don’t know what to do.*” (Asylum-seeking woman from Mexico, 23 years old)

Another woman described how this interacted with anti-Black racism and xenophobia, with her husband being turned away from renting an apartment because he was Haitian.

*“The lady who... owned a house knew that a man was coming to see the house and when my husband arrived, the lady was at the door. She saw that a Haitian was coming and closed the door. She closed it. So she won't rent the house to Haitians*.” (Asylum-seeking woman from Haiti, 40 years old)

Due to the lack of available spaces in shelters, many women needed to find their own place to live. With little available housing stock and the record numbers of migrants waiting at the border, affordable housing was nearly impossible to find. With their financial precarity, many women found themselves limiting food so that they could pay for rent, or were forced to remain in exploitative or unsafe situations (e.g., staying with abusive partners) in exchange for shelter.

Asylum deterrence policies in place since March 2020 led to women being trapped within the cycles of GBV with no escape, no access to security or safe spaces. By migrating, women had expected to get away from the violence in their home countries and the reasons that they had initially decided to flee. However, many found that Tijuana was not the safe haven for which they had hoped and, due to limited access to the asylum-seeking process in the U.S., they were stuck in these unsafe spaces, perpetuating their trauma with no foreseeable resolution. One woman described how she and her family had been fleeing a cartel in their home country, but they realized that this cartel was also present in Tijuana.

“[*We will seek asylum in the U.S.] if we can, yes. To cross over at once. Because now we realize that the [name of drug organization] arrived here and I don't know how it is here, but we are afraid that it will be the same as [in our place of origin].”* (Asylum-seeking woman from Mexico, 26 years old)

Although some women described finding supports through religious or social agencies, in many cases women felt they had no choice or autonomy regarding where they received supports and were forced to place their trust in whoever offered help. It was clear that the increased vulnerability of asylum-seekers engendered by asylum deterrence policies provided the opportunity for perpetrators of violence and other harms to take advantage of their desperate situation.

### Mental and physical health consequences of GBV across the migration journey

Women reported that their exposures to GBV were significant and long-lasting, including both mental and physical harms. Most women described living in constant fear, which affected their freedom to move freely about their neighborhoods and to live in peace. While many women did not label their experiences as “traumatic”, perhaps due to the pervasiveness of GBV in their lives, many described experiences and symptoms of trauma linked to the physical and sexual violence and otherwise dangerous situations that they experienced or witnessed around them.

*“Well, me, when I arrived and got off the bus, I was scared because I didn't know the street or where I should go. And since it is dangerous in the streets, you do not know what could happen. You could be mugged, or they could take away the baby. And I was careful, and I arrived all the way there*” (Asylum-seeking woman from Mexico, 19 years old)

“*But the fear is the same. It is not our country. We do not know each other well. We do not know. But I think you live that even yourselves. We don't know when a person is good, or if they are bad. There is always that fear*.” (Asylum-seeking woman from Honduras, 26 years old)

*“In the streets, yes, I am very, very afraid. Here [in Tijuana], I am very, very afraid. I suffer more from stress. Because one time when my husband left his work, he encountered thieves… And that’s why I’m very afraid here. That’s why when I don’t have to go out, I stay at home with the children… I live in fear.” (Asylum-seeking woman from Haiti, 40 years old)*

These quotes highlight the constant state of vigilance in which asylum-seeking women live, as well as the social isolation that it brings for themselves and their children, as they do not feel as though they can trust others or the world around them.

Women’s personal experiences of trauma were compounded by the loss and grief of losing others along the journey, whether these people had been victims of violence or were individuals with whom they had lost contact and feared for the worst. One woman shared her experience crossing the Darién Gap, which is a particularly dangerous region along a migration pathway between Colombia and Panama and the only overland path connecting Central and South America: “*there is a lot of death on the road because some people are already tired, they can’t, they can’t get to their destination*” (Asylum-seeking woman from Haiti, 32 years old). Many women experienced trauma stemming from structural violence by immigration and other government agencies, including forced displacement and disappearance of family members. For example, one woman recounted her experience at an encampment at the Chaparral point of entry, which was torn down by municipal authorities. Within one day, the woman and her families were separated from other migrating families with whom they had become close, who had disappeared suddenly in the night. Such experiences disrupted important social support networks, and not knowing what had occurred or where friends, family, or community members had gone, or whether they were safe or not, was a constant source of stress for the women, and impacted perceived safety for themselves and their families.

“*There was a lot of sadness [after we left El Chaparral] because some of our friends and colleagues who were there, we never heard from them again. What happened to them?... They disappeared…”* (Asylum-seeking woman from Honduras, 36 years old)

Trauma was often unspoken. In our interviews, it became apparent that many women did not disclose all of the trauma that they had experienced, perhaps because they did not want to relive these or because the experiences were too frequent to fully recount. When asked about any experiences of discrimination related to migration status when interacting with health or social services, one woman stated that she “*didn’t include any of that. I didn’t even include the abuses that happened, none of that…It gives me … I don’t know what, to be reliving so many things*” (Asylum-seeking woman from Honduras, 26 years old).

### Lack of access to responsive justice and violence-related supports

Limited supports were available for addressing GBV through criminal legal actions against perpetrators or for supporting women who had experienced GBV, which allowed perpetrators of violence to operate with impunity, further perpetuating vulnerability to GBV for women in our study.

Because of limited options to meet basic needs, many women were forced to live with daily threats of GBV and were often limited in their ability to seek recourse for sexual or economic coercion. One woman described her experiences of these coercions with an extended family member who was providing housing for her and her husband, which she felt forced to endure to ensure that she and her family had a “safe” place to sleep. She reported this abuse to her husband but, because they had no other option for shelter, this was seen as a necessary cost of shelter and the abuse was never reported or addressed (e.g., to police).

*“They started to demand that [my husband and I] pay them money… that nothing is free… And then came harassment from [the extended family member] towards me… He texted me and asked me how much I would charge him to be with him. And I got very scared and I told my husband. But since we were in his house, [my husband] didn’t want to say anything because if he kicked us out, we were not going to have a place to go*.” (Asylum-seeking woman from Mexico, 18 years old)

Some women attempted to report their experiences of GBV in their home countries and in Mexico, including Tijuana, but none were offered any meaningful investigation or recourse. These stories were indicative of the acceptance of GBV as a regular, normal, and allowable occurrence for women, particularly for racialized, migrant women in transit.

*Over there, they don’t do anything [regarding the threats], they do nothing. They do not help us. One has to flee.”* (Asylum-seeking woman from Mexico, 24 years old)

Other women were silent about their experiences of GBV, perhaps because of previous experiences in which reporting failed to result in any meaningful improvements or justice, because of fear, or because they had internalized the normalization of GBV in their lives. One woman stated that "*you need to be silent”* and another had not disclosed her experience of sexual assault during migration to anyone prior to their interview. Importantly, none of the women in our study reported seeking or receiving any formal support for themselves to mitigate the negative physical and mental health impacts of GBV in their lives (e.g., trauma-informed mental health supports).

Women’s knowledge and first-hand experience that there was no available recourse for the GBV they experienced and no support for the trauma they lived with daily highlighted the perpetuation of GBV in their lives. The legal violence of asylum deterrence policies holding them in limbo at the Mexico-U.S. border acted to limit women’s autonomy and reproductive rights, depriving them of an escape from the constant fear and threat of GBV and of a legal status that might protect them from their perpetrators.

## Discussion

This qualitative study presents the lived experiences of pregnant and parenting asylum-seeking women at the Mexico-U.S. border in the context of structural and legal violence through continuing asylum deterrence policies and the implementation of Title 42 during the COVID-19 pandemic, which represents one of the most restrictive border control measures implemented in recent U.S. history. We documented that pregnant and parenting asylum-seeking women routinely faced multiple forms of GBV perpetuated by asylum deterrence policies at all stages of migration (pre-migration, in transit, and in Tijuana). Indefinite wait times to cross the border and inadequate/unsafe shelter exacerbated further vulnerability to GBV and represented a form of “entrapment” processes [40]. Repeated exposure to GBV contributed to poor mental health, and women faced severe gaps in violence supports and justice in Tijuana. These findings collectively highlight the ways in which current U.S. asylum deterrence policies operate as pervasive forms of structural and legal violence that perpetuate and exacerbate pregnant and parenting asylum-seeking women’s vulnerability to and risk of GBV during the asylum-seeking process, ultimately constraining the right to access asylum and interacting with other forms of violence including racism, sexism, and xenophobia. To our knowledge, this is one of few empirical studies addressing this critical topic within the context of asylum deterrence policies at the Mexico-U.S. border during the COVID-19 pandemic.

This study aligns with previous research documenting the high levels of GBV among asylum-seeking women across their migration journeys [11, 41] related to structural and legal violence from racist, colonial, and patriarchal systems, and extends it to focus on pregnant and parenting asylum-seekers at the Mexico-U.S. border in the context of asylum deterrence policies. Other studies have acknowledged the “normalization of GBV” among migrant women [42] and the ubiquity of GBV along migration pathways [15, 43, 44], particularly in the context of highly militarized and fortified borders and within exclusionary immigration policies, similar to protracted conflict in other global settings and along other established migration routes [45–48]. These experiences of violence may be normalized or accepted by migrant women as the “price to pay” but may also be used in their migration narratives to reinterpret their own stories and futures, reframing the idea of “victimization” and “vulnerability” of asylum-seeking and migrant women experiencing violence [48, 49]. This supports our findings, both of our participants’ vast exposures to GBV during their migration journeys and that systems and policies around migration perpetuate structural and legal violence against women by creating conditions that allow the frequent and pervasive GBV to continue without accountability and ignore its long-lasting impacts.

Our study is one of the first to examine GBV within the context of asylum deterrence policies for pregnant and parenting asylum-seeking women at the Mexico-U.S. border since the beginning of the COVID-19 pandemic. Although several recent reports from frontline agencies supporting migrants at the Mexico-U.S. border have demonstrated how U.S. asylum deterrence policies increase migrant women’s vulnerability to GBV [13, 19, 50, 51], few research studies have examined the impact of immigration policy on the structural perpetuation of GBV and instead, continue to focus on individual- and interpersonal-level factors. For example, instead of recognizing the role of unresponsive, discriminatory, and corrupt systems in the lack of repercussions for perpetrators of GBV, a critical interpretative synthesis of 84 studies examining migrant experiences of sexual and GBV found that the reason women do not report their experiences is related to internal stigmatizations of being a victim of GBV [5]. There is a clear need for more research to investigate, at a structural level, the marginalizing impact of asylum deterrence policies on pregnant and parenting women’s experiences of GBV, trauma, and mental health.

Our study adds to the growing body of literature documenting the negative impact of U.S.-based asylum deterrence policies on experiences of GBV for asylum-seeking women, particularly related to trauma. For example, a 2021 critique of Asylum Cooperative Agreements between the U.S. and the Northern Triangle countries, which permitted the expedited removal of asylum seekers from the U.S. (i.e., a transit ban), found that asylum-seeking women had high rates of exposure to traumatic violence, with 91.4% reporting repeated trauma exposures related to physical assault [52], sexual assault, threats of violence, and witnessing violence [53]. Another study analyzing asylum-seekers’ affidavits after living through the Migrant Protection Protocols (MPP) found that the MPP increased asylum-seekers’ risk of re-traumatization and affected their ability to heal from pre-migration trauma [54], perpetuating the harms of GBV [54]. Other studies have highlighted the need for mental health supports in asylum-seekers, given the high levels of trauma exposure, both before and after crossing the border into the U.S [6, 55–57].

In alignment with other humanitarian and human rights advocates [13], the results of this study highlight the clear need for the swift removal of asylum deterrence policies in the U.S. Although Title 42 expired in May 2023, it has since been replaced with other restrictive immigration policies such as the use of the CBP One App and the Transit Ban which continue to deter asylum-seeking by limiting the opportunities to seek asylum through official measures and placing harsh punishments on those who attempt to circumvent these measures [22, 23, 58–60]. It is clear that these policies and practices will continue to have impacts on asylum-seekers’ rights and their well-being while they continue to wait in border cities such as Tijuana. Furthermore, inconsistent application of “options” for entry to the U.S. and extreme backlog for processing, such as humanitarian parole for asylum-seeking pregnant women, limits the effectiveness of such policies and negates their potential value for those intending to apply due to pregnancy. Even when humanitarian parole is able to be processed in an appropriate timeframe, the discretionary nature of humanitarian parole applications means that this pathway to entry into the U.S. is arbitrary, and pregnant asylum-seekers continue to experience mistreatment and detention at the hands of U.S. border officials [61]. Changes to the asylum-seeking process to enhance its usefulness would help to reduce exposure to the structural, legal, and gender-based violence experienced by pregnant and parenting asylum-seekers at the Mexico-U.S. border.

There is also the need for scaled-up investment of legal, social, and humanitarian resources available to pregnant and parenting asylum-seekers. Although research from other humanitarian contexts has identified the importance of access to GBV services after women have crossed the border into safety [62, 63], little has been done in the U.S.-Mexico border spaces where asylum seekers have been forced to wait in unsafe spaces indefinitely, where they have limited access to supports or legal recourse for their experiences of GBV. Our research highlights the need for appropriate, timely, and accessible options for reporting GBV and for holding perpetrators accountable. Trauma-informed supports around GBV are also needed for pregnant and parenting asylum-seeking women in transit and throughout their resettlement in the U.S. Other studies have shown the importance of providing access to services for GBV for refugee and asylum-seeking women in their destination countries; however, due to the lack of humanitarian aid given to asylum-seekers waiting at the Mexico-U.S. border, these services were not available or accessible to women in our study and thus, there is a need for significant investment to support asylum-seeking women even before they cross into the U.S.

While the current paper focuses on pregnant and postpartum asylum-seeking women’s experiences of violence, these women also faced significant threats to their own health and the health of their children during and after pregnancy due to this lack of access to care, and the intersecting impacts of structural and legal violence. A 2018 systematic review of systematic reviews on perinatal health outcomes and care among asylum-seekers and refugees found that perinatal outcomes are worse among migrant women, especially mental health, maternal mortality, preterm birth, and congenital anomalies, and that access to care was limited by multiple factors at the interpersonal, organizational, community, and structural levels [64]. Furthermore, given that babies born in Mexico are granted Mexican citizenship and provide the potential for parents and siblings of that child to apply for permanent residency in Mexico (although accessibility and affordability of this process remain issues for many families), the birth of a child while waiting in Tijuana could impact future asylum-seeking claims in the United States. Future research should is needed to examine the impacts of structural, legal, and gender-based violence on the perinatal outcomes for pregnant and post-partum asylum-seekers and the impact of these experiences on their migration trajectories.

This study has both strengths and limitations. Using rigorous, community-based, qualitative research methods, we document the first-hand experiences of pregnant and parenting asylum-seeking women trapped at the Mexico-U.S. border, adding urgency to the call for the removal of asylum deterrence policies. Our qualitative interviews provide insight into the diverse experiences of pregnant and parenting asylum-seekers since the beginning of the COVID-19 pandemic amidst the implementation of Title 42. Although our study was provided rich data on these women’s experiences in the context of Title 42, as important to note that the immigration policy landscape is ever-changing. While new constellations of asylum deterrence policies are anticipated to have similar impacts, this represents an important area for future and ongoing research. Finally, despite our attempts to capture a diverse sample, our study may not have fully captured the full severity of GBV faced by asylum-seeking women at the Mexico-U.S. border. Women who experienced more severe forms of GBV, such as death, kidnapping, or human trafficking enroute to Tijuana would not have been part of our study sample. As well, as our sample focused on the experience of pregnant and parenting asylum-seeking women, it is possible that our results may not extend to women who are not pregnant or parenting.

## Conclusion

In this qualitative study, the narratives of pregnant and parenting asylum-seeking women in Tijuana demonstrate the ways in which current asylum deterrence policies perpetuate and exacerbate vulnerability to and risk of GBV during the asylum-seeking process. There is an urgent need to end U.S. asylum deterrence policies and to provide respectful, appropriate, and adequately resourced humanitarian supports to this population, with the ultimate goal of reducing women’s risk of GBV and trauma. Additionally, both appropriate and timely access to meaningful justice for survivors of GBV and targeted and trauma-informed supports around GBV for pregnant and parenting asylum-seeking women at the Mexico-U.S. border are recommended.

## Data Availability

The data that support the findings of this study are not publicly available due to the sensitive and potentially identifiable nature of the data, but excerpts of the transcripts relevant to the study fundings are available within the manuscript.

## Footnotes

1 Given that the eligibility criteria defined participants as having been pregnant or postpartum since the beginning of the COVID-19 pandemic (March 2020), but data collection was only done in 2022, many of the women were no longer in the postpartum period and thus, for the rest of the article, we refer to our study population as “pregnant and parenting asylum-seeking women”.
